# Zonulin: A Potential Marker of Intestine Injury in Newborns

**DOI:** 10.1155/2017/2413437

**Published:** 2017-07-09

**Authors:** Anna Tarko, Anna Suchojad, Marta Michalec, Małgorzata Majcherczyk, Aniceta Brzozowska, Iwona Maruniak-Chudek

**Affiliations:** ^1^Department of Intensive Care and Neonatal Pathology, School of Medicine in Katowice, Medical University of Silesia, Katowice, Poland; ^2^Health Promotion and Obesity Management Unit, Department of Pathophysiology, Faculty of Medicine in Katowice, Medical University of Silesia, Katowice, Poland

## Abstract

**Introduction:**

Zonulin (ZO), a new diagnostic biomarker of intestinal permeability, was tested in newborns presenting symptoms of infection and/or inflammation of the gut or being at risk of intestinal pathology.

**Material and Methods:**

Serum ZO was assessed in 81 newborns diagnosed with sepsis, necrotizing enterocolitis (NEC), rotavirus infection, and gastroschisis, also in extremely low gestational age babies, and in controls (healthy newborns). ZO concentration was compared to C-reactive protein (CRP) and procalcitonin (PCT) values, leucocyte and platelet count, basic demographic data, and the value of the Neonatal Therapeutic Intervention Scoring System (NTISS).

**Results:**

Median values of ZO were markedly higher in groups with rotavirus infection and gastroschisis (36.0 (1-3Q: 26.0–43.2) and 20.3 (1-3Q: 17.7–28.2) ng/ml, resp.) versus controls (3.5 (1-3Q: 2.7–4.8) ng/ml). Its concentration in the NEC group was twice as high as in controls but did not reach statistical significance. ZO levels were not related to NTISS, CRP, and PCT.

**Conclusions:**

Zonulin is a promising biomarker of intestinal condition, markedly elevated in rotavirus infections. Its role in defining the severity of necrotizing enterocolitis and the risk for perforation is not well described and needs further evaluation. An increase in zonulin may not be parallel to the release of inflammatory markers, and low CRP should not exclude an injury to neonatal intestine.

## 1. Introduction

Gastrointestinal tract is an important organ which is attributed not only to digestion and absorption of nutritional compounds but also to immunologic function, modulation of inflammatory response, and triggering of autoimmune diseases. Complexity of morphological structure of the gut is not completely revealed, and new findings show the broader meaning and significance of this organ. Particularly, in neonatal period and infancy, we are still discovering the importance of the gut in overall wellbeing. Newborns and infants are undergoing developmental changes and processes, including immunity and managing inflammatory response. The less mature newborn the more fragile tissues of gastrointestinal track appear to be predominantly in extremely low gestational age premature infants. Additionally, some illnesses may have particularly severe clinical course in this period of life, such as necrotizing enterocolitis (NEC) or sepsis (especially caused by Gram-negative species). They are both known for their poor outcome in most of the cases, and early symptoms are not specific and do not indicate prognosis (including intestinal perforation or septic shock with multiorgan dysfunction). Also, some frequent and widespread illnesses like rotavirus infection, in very rare cases, are followed by bacterial sepsis, possibly related to intestinal bacterial translocation. A search for a sensitive biomarker of intestine injury is ongoing, as the traditional parameters of inflammatory status are not sufficiently predictable.

Zonulin (ZO), as an established indicator of intestinal permeability, has been described more than 20 years ago [1, 2]. It is claimed to have a potential value in delineating the mechanism of paracellular “gateway” in the intestines [3–5]. This 47 kDa single protein is isolated from a membrane complex (claudin-occludin-guanylate kinase-like proteins ZO-1, ZO-2, and ZO-3) forming tight junction (TJ) in the apical part of intestinal endothelium [6]. ZO has also been known as pre-HP2, due to its identity to the precursor of haptoglobin-2 [7]. It is one of the main factors securing the adequate action of the “gate of the gut” mechanism by reversibly influencing TJ's tightness [8, 9]. Incorporation of proteinase-activating receptor 2 (PAR2) and epidermal growth factor receptor (EGFR) allow to cross the intestinal barrier for molecules exceeding approx. 3.5 kDa [7]. Experimental studies revealed that the rise in ZO concentration is parallel to increased intestinal permeability [6]. Further, clinical investigations showed that patients suffering from entities known for component of low-grade inflammation (such as type 2 diabetes, celiac disease, and obesity) presented increased ZO concentration [10–13]. The same is true for diseases of autoimmunological pathomechanism (Crohn's disease, type 1 diabetes) [6, 14]. ZO increase and disruption of TJ has also been demonstrated in an animal model of NEC with intestinal TJ destruction proven by immunohistochemical evaluation [15].

Based on this findings, we decided to measure serum ZO concentration in newborns suffering from entities typical for this population, focusing on prematurity, NEC, sepsis, and rotavirus infection. The aim of our pilot study was to check if there is any correlation between ZO level as a marker of intestinal permeability and concentration of inflammatory markers and also the general clinical condition of our study group.

## 2. Material and Methods

### 2.1. Patients

81 newborns admitted to Intensive Care and Neonatal Pathology Department of paediatric university hospital were enrolled to this observational study. All of the patients were outborn, referred to the unit from first, second, or third level nurseries because of diagnosed pathological symptoms, or they were admitted from home through the hospital emergency unit due to sudden illness. The patients were diagnosed and treated according to the established ward protocols, and this retrospective project did not interfere with the course of medical management. Demographic and clinical data were achieved from patients' files, and ZO evaluation was performed using serum samples saved after planned laboratory tests. Severity of clinical condition and intensity of medical management were evaluated using the Neonatal Therapeutic Intervention Scoring System (NTISS).

Bioethical Committee of Medical University of Silesia gave its consent for using the remaining biological material after standard workup. The control group (*N* = 14) consisted of newborns not diagnosed finally with infection or any other condition causing inflammatory process. The sepsis group included 12 term newborns suffering from early to late severe sepsis. They were seriously ill, suffering from respiratory and, in some cases, circulatory insufficiency, treated with at least two broad-spectrum antibiotics and in 4 cases receiving catecholamines in intravenous infusions. The ELGAN group consisted of extremely low gestational age newborns (ELGAN) on their first days of life, hospitalized for suspected intrauterine infection, requiring mechanical ventilation, total parenteral nutrition (TPN), and constant monitoring of basic biophysical parameters. The NEC group included 2 term, 2 near-term, and 8 preterm babies diagnosed with necrotising enterocolitis. In 5 cases, perforation occurred during the course of an illness. They were all intensively treated with respiratory and circulatory support, antibiotics, and TPN. In term babies and one late preterm, the occurrence of NEC was combined with congenital heart malformations causing poor peripheral perfusion. The other late preterm baby presented symptoms of NEC in the course of severe sepsis. The rotavirus group was formed of term newborns who were diagnosed with rotavirus infections. General clinical condition was stable; they did not require any respiratory or circulatory support and were enterally fed and given additionally intravenous infusion. The last group, abdominal wall defect (AWD), included only 3 patients who were diagnosed with gastroschisis and prepared for surgical closure. All serum samples were analyzed only once in each patient, focusing on the day of presenting clear clinical symptoms of the disease.

### 2.2. Laboratory Measurements

Serum zonulin measurements were performed using a commercially available ELISA kit (Immunodiagnostic AG, Bensheim, Germany). Mean intracoefficient of variance was less than 5%, and sensitivity specified below 0.01 ng/ml.

### 2.3. Data Analysis

Sepsis was defined as systemic inflammatory response syndrome (SIRS) with evidence of infection (positive microbiological culture, clinical symptoms). Severe sepsis was identified based on clinical and laboratory parameters with symptoms of at least two organ or system dysfunctions.

Routinely used immunological test detecting rotavirus antigen in a stool was used for the diagnosis of rotavirus infection in symptomatic newborns.

NEC was recognized based on radiologic and ultrasound findings in newborns with risk factors, presenting signs and symptoms suggesting this entity. Perforation was confirmed by X-ray imaging, and patients were qualified for surgery.

In all cases of gastroschisis, the patients were operated in a one-stage manner (underwent primary closure). Serum samples acquired from the last mentioned group were secured before the surgery.

### 2.4. Statistical Analysis

Analyses were performed using the STATISTICA 10.0 software (StatSoft, Tulsa, OK, U.S.). Normality of distribution was tested with the Kolmogorov-Smirnov test. The data presented are median values with 1 and 3 quartiles (1-3Q) due to nonnormal distribution of the variables. For comparison of groups, we used the *χ*^2^ test (qualitative variables) and ANOVA or Kruskal-Wallis one-way analysis of variance by ranks followed by the Mann–Whitney *U* test (quantitative variables). Correlation coefficients were calculated according to Spearman. *p* values < 0.05 were considered as statistically significant.

## 3. Results

Demographic and clinical characteristics of the study population are presented in Table 1 separately for each group of patients. Significantly lower body weight at the time of evaluation and gestational age values were seen in the NEC and ELGAN groups; the last one was also described by lower 1st and 5th minute Apgar score, as could be expected. Newborns suffering from rotavirus infection were significantly older (24 and 17–25 days of life, median and 1-3Q, resp.). Median CRP values were highest in the group of term newborns with sepsis and in patients with NEC (52.1 (1-3Q: 15.8–94.1) and 22.8 (1-3Q: 6.1–59.1) mg/dl, resp.), while PCT values were also elevated in septic newborns and in extremely low gestational age babies (9.0 (1-3Q: 2.2–14.2) and 5.7 (1-3Q: 2.7–10.6) ng/ml, resp.). We cannot exclude that the elevation of PCT in the group of premature babies was due to the physiological rise in PCT in the first 48 hours of life. Decrease in platelet count was noticeable in the ELGAN group with median 133 × 10^3^/*μ*l, but not in NEC patients, in whom this pathology was more frequently expected.

Analysis of serum ZO concentration revealed statistically higher median values in the rotavirus infection group and gastroschisis patients (36.0 (1-3Q: 26.0–43.2) and 20.3 (1-3Q:17.7–28.2) ng/ml, resp.). Median ZO concentration in the NEC group was more than twice as high as in controls, but the difference did not reach statistical significance due to the low number of study cases.

We could not find any correlation between ZO values and parameters of inflammatory response (CRP, PCT) (Table 2). A low platelet number as well as some demographic parameters (1st and 5th minute Apgar score, body weight) correlated with ZO concentration (Table 2).

## 4. Discussion

Our results suggest that ZO is a valuable biomarker of intestinal injury. We showed that ZO concentration is markedly increased in newborns with rotavirus infection and to a less extent in patients with gastroschisis and those suffering from NEC. The study data consist of an indirect evidence for increased intestinal permeability. Certainly, rotavirus infection is a clinical model of massive injury to the intestinal epithelium that has to be followed by increased permeability [16]. Clinically interesting, but unanswered question is whether an injury to TJs, pictured by elevated ZO serum concentration, could be considered as a biomarker of risk for intestinal bacterial translocation even without perforation. Our rotavirus infection group, despite showing the highest values of ZO, did not present severe deterioration of clinical condition in septic mechanism. We could not find any published paper on ZO and rotavirus infection, but there was one regarding enteroviruses. Vorobjova et al. [17] reported correlations between an intensity of mucosal atrophy in small bowels and increased ZO serum levels (comparable to ZO concentrations in our rotavirus group) with a higher density of enteroviruses.

We checked the course of disease in the individuals representing the NEC group and found that ZO values were not higher in patients with perforation. Therefore, we suggest that increased ZO level reflects more the extent (area) than the severity of intestinal injury. We would not however dare to draw any strong conclusions, as the study group of patients with NEC was small and our project was not designed as a prospective observation. Nevertheless, the idea of a new potential biomarker of intestinal wall injury seems to be very promising.

A search of medical databases is providing just few papers on the topic of ZO in paediatric population. Recently, a mild increase in ZO and proinflammatory cytokines was revealed in infant colic, that is, suspected to be at least partially caused by low-grade systemic inflammation [18].

A problem of microinflammatory injury to the intestinal barrier is raised in patients with type 2 diabetes and obesity [10, 19]. Much more severe intestinal injury occurs in patients with Crohn's disease [6]. In all these entities, elevated ZO was detected along with increased inflammatory markers (CRP, PCT, IL6, and TNF-*α* among others). Therefore, increased ZO level may possibly be considered as another inflammatory parameter. However, in our study, we showed no correlation between ZO and proinflammatory markers. We cannot deny yet that longer observation may have changed this relation. An alternative explanation for our findings is that ZO level reflects more interstitial injury than inflammation generated by the injury. This interesting hypothesis necessitates further studies.

Observations made in NEC patients characterised by relatively low values of ZO in comparison to newborns with rotavirus infection are in line with our suggestions. NEC patients showed high NTISS punctuation and markedly elevated CRP and PCT, which all together fit in a clinical picture of severely ill newborns, mostly premature ones. The group included individuals with perforation and severe sepsis, but ZO concentrations were much lower than those in the rotavirus group. The last ones did not have any indicators of severe inflammation, and the course of disease was stable and uncomplicated.

We are aware that NEC is a multifactorial entity and many components decide on the final clinical presentation. Intestinal microbiota, including the number and diversity of bacterial species, antibiotic usage, feeding regimen, and the kind of nutritional support (human milk versus formula) are important explanatory features in NEC occurrence, severity of disease course, and prognosis [20, 21]. Many positive opinions are stated about probiotics and its protective role against NEC development and severity, possibly by decreasing inflammatory response, including proinflammatory cytokine secretion [15]. Very likely, there are other factors of significant influence on disease outcome, and one of them could be congenital heart malformation causing the decrease in peripheral perfusion, leading to tissue necrosis and NEC as consequences. In such situation, a bacterial causative role in pathogenesis of NEC might be diminished. Also, the marked differences in response to NEC, presented by small intestines and colon, cannot be excluded as meaningful factors in final prognosis [20]. All this emphasized that many aspects need to be taken into consideration in analyzing the relation between ZO levels and clinical picture of NEC. Our observations were not supported by all the necessary data and the results we attained in NEC patients rise many questions regarding intestinal epithelium and mechanisms of intestinal necrosis, and at this point, conclusions would be premature.

Our data acquired from septic patients again were not quite expected. The development of septic SIRS is strongly related to the gut and dysfunction of the intestinal wall, with a crucial role of epithelial TJs and intestinal permeability, leading to increased proinflammatory activity [4, 22, 23]. This is consistent with the results published by Klaus et al. [24] who measured ZO concentration in adult septic patients and found its values elevated in comparison to nonseptic individuals. However, there were no differences in ZO levels between septic cases of abdominal origin and those of other ethiopathogenesis. Again, according to Deitch [25] and Faries et al. [26], also burns or trauma were causing an increase in intestinal permeability. These findings would suggest that ZO release from TJs and intestinal permeability are induced by a “causative” factor(s) stimulating these processes indirectly. Proinflammatory factors seem to be a good explanation. That is why we actually suspected the simultaneous increase in inflammatory parameters (CRP, PCT, and leucocytes) in good correlation with ZO levels, but our results did not prove it. Similarly, Klaus et al. did not demonstrate any correlation between ZO and inflammatory markers [24]. Another author observed that perioperative probiotic treatment was beneficial for patients, reducing the rate of infections, decreasing ZO levels and intestinal permeability (bacterial DNA measurement), and reducing the time of antibiotic treatment [27]. This suggests that the composition of intestinal microflora and possibly the proportions of “good” and “bad” bacteria matters. Unfortunately, we did not have the data regarding intestinal bacterial transfer in sepsis, so we were not able to compare these results. It would also be difficult to discuss the differences in ZO levels, as our septic patients presented rather low values. The reason for this may lie in their general condition, not severe enough to show spectacular rise of the marker. However, to finally evaluate ZO role in sepsis, we should check its value also in very rare but severe cases, when peripheral perfusion is almost stopped, peristalsis is paralysed and risk of intestinal injury rises significantly. The other thing is to follow ZO levels for several days during sepsis. Longer observational period may reveal changes in ZO which were not noticed in one-point measurements.

We did not have many opportunities to compare ZO levels in extremely premature infants with values in other patients. Saleem et al. [21] observed 43 newborns below 33 weeks of gestation, including 12 equal or lower than 28 weeks and measured ZO, alpha-1-anti-trypsin, and the urinary lactulose/rhamnose ratio. They found no correlation between ZO and the lactulose/rhamnose ratio and concluded that intestinal barrier function was impaired in that group of patients and maturation depended on gestational and postnatal age. What is more, according to Saleem et al., ZO was not involved in TJ maturation. This could be an explanation for our results, but the lack of absolute ZO values in a cited article withdraws us from making comparisons. Analyzing the ELGAN group, we were not surprised that CRP was not high (frequently seen in less matured newborns) and we analyzed PCT levels with caution, keeping in mind its peak value on the first two days of life. A low number of platelets were in parallel to generally serious clinical condition, confirmed by high NTISS evaluation.

Newborns suffering from gastroschisis created a group with a very small number of patients, and it prevented us from drawing more sophisticated statistical descriptions. Nevertheless, we noticed particularly high ZO concentration, even though the newborns' intestines were not injured by squeezing, torsion, introduction of feeding, or invasive procedure before and at the time of blood sampling.

Talking about ZO as a marker of intestinal permeability, we did not prove the disturbances in intestinal wall “leakage” by golden standard, which means enteral administration of unabsorbed substance followed by its measurement in urine samples. A mixture of lactulose and rhamnose disaccharides is currently recommended for this test, but evaluation of urine samples is technically demanding [21]. Clinically more important is the fact that prematures suspected for NEC or newborns in septic shock are not allowed to consume any enteral substances, especially when it comes to just about research and not proved medication.

This pilot study indicates the urgent need for further evaluation of intestinal condition in typical neonatal entities and search for marker or panel of markers enabling to identify individuals at risk of bacterial translocation followed by severe sepsis or intestinal perforation.

Summarizing the results, the following conclusions can be proposed:

1. Zonulin is a promising biomarker of intestinal condition, possibly an injury, and is markedly elevated in rotavirus infections in newborns.
2. Its role in defining the severity of necrotizing enterocolitis and risk for perforation is not well described and needs further evaluation.
3. An increase in zonulin may not be parallel to the release of inflammatory markers, and low CRP should not exclude an injury to neonatal intestines.

## Figures and Tables

**Table 1:** Demographic and clinical characteristics of the study group (*N* = 81) with distribution according to clinical diagnosis—median values and 1–3 quartiles.

	Control group	Sepsis group	ELGAN group	NEC group	Rotavirus group	AWD group
*N*	14	12	24	12	16	3
Gender (F/M)	6/8	2/10	8/16	8/4	5/11	2/1
Birth weight (g)	3260 (2850–3440)	3208 (2505–3465)	1120^∗∗∗^ (800–1400)	1955^∗∗∗^ (1100–2470)	3000 (2495–3690)	2250 (2100–2700)
Body weight (g)	3260 (2850–3400)	3208 (2505–3465)	1120^∗∗∗^ (800–1400)	1962^∗∗∗^ (1115–2585)	3302 (2790–3805)	2270 (2190–2610)
1'Apgar (pts)	9 (8–10)	9 (7–10)	5^∗∗∗^ (3–6)	6 (5–8)	10 (8–10)	6 (2–8)
5'Apgar (pts)	10 (9-10)	10 (7–10)	5^∗∗∗^ (4–7)	8 (7-8)	10 (9-10)	7 (7-8)
GA (wks)	39 (37–39)	38 (36–40)	28^∗∗∗^ (25–31)	33^∗∗∗^ (29–36)	38 (36–39)	37 (36–38)
DOL	5 (2–11)	10 (3–18)	2 (1–5)	7 (4–25)	24^∗∗∗^ (17–27)	3 (2–5)
MOD (SD/CS)	9/5	8/4	6/18	3/9	7/9	0/3
Zonulin (ng/ml)	3.5 (2.7–4.8)	3.5 (2.8–4.3)	3.8 (2.0–5.4)	8.6 (3.8–11.9)	36.0^∗∗∗^ (26.0–43.2)	20.3^∗^ (17.7–28.2)
NTISS (pts)	3 (2–5)	18^∗∗∗^ (13–26)	24^∗∗∗^ (21–26)	23^∗∗∗^ (18–25)	8 (7–9)	21^∗∗^ (19–23)
CRP (mg/dl)	0.2 (0.2–1.6)	52.1^∗∗∗^ (15.8–94.1)	3.1 (0.9–9.5)	22.8^∗^ (6.1–59.1)	0.7 (0.2–2.1)	47.5 (1.9–58.2)
PCT (ng/ml)	0.1 (0.1–0.2)	9.0^∗∗^ (2.2–14.2)	5.7^∗^ (2.7–10.6)	2.2 (0.7–12.0)	0.1 (0.1–0.3)	2.1 (0.9–3.3)
WBC (^∗^1000/*μ*l)	11.7 (9.9–13.0)	10.8 (5.7–21.0)	16.6^∗^ (9.6–23.1)	13.3 (11.3–19.8)	10.8 (8.1–115)	10.3 (7.6–24.2)
PLT (^∗^1000/*μ*l)	277 (185–322)	174 (86–258)	133^∗∗^ (92–187)	226 (166–359)	386^∗∗^ (310–484)	230 (213–263)

ELGAN: extremely low gestational age newborn; NEC: necrotizing enterocolitis; AWD: abdominal wall defect; *N*: number; F: female; M: male; g: grams; pts: points; GA: gestational age; wks: weeks; DOL: day of life; MOD: mode of delivery; SD: spontaneous delivery; CS: cesarean section. Statistical significance versus control group. ^∗^*p* < 0.05; ^∗∗^*p* < 0.01; ^∗∗∗^*p* < 0.001.

**Table 2:** Correlation coefficients (by Spearman) between zonulin levels and selected demographic and clinical parameters.

	R (Spearman)	*p* value
WBC	−0.215	NS
PLT	0.448	<0.001
NTISS	−0.186	NS
CRP	−0.100	NS
PCT	−0.101	NS
GA	0.196	NS
1'Apgar	0.313	0.007
5'Apgar	0.289	0.01
BW	0.316	0.004

WBC: white blood count; PLT: platelets; NTISS: Neonatal Therapeutic Intervention Scoring System; CRP: C-reactive protein; PCT: procalcitonin; GA: gestational age; BW: body weight.
